# The adherence-associated Fdp fasciclin I domain protein of the biohydrogen producer *Rhodobacter sphaeroides* is regulated by the global Prr pathway

**DOI:** 10.1016/j.ijhydene.2020.07.108

**Published:** 2020-10-16

**Authors:** E.-L. Jeong, S.J. Broad, R.G. Moody, M.K. Phillips-Jones

**Affiliations:** a Astbury Centre for Structural Molecular Biology, Faculty of Biological Sciences, University of Leeds, Leeds, LS2 9JT, United Kingdom; b Department of Molecular Biology & Biotechnology, University of Sheffield, Sheffield, S10 2TN, United Kingdom; c National Centre for Macromolecular Hydrodynamics, School of Biosciences, University of Nottingham, Sutton Bonington, Leicestershire, LE12 5RD, United Kingdom

**Keywords:** *Rhodobacter sphaeroides*, Biohydrogen, Green energy, Prr two-component system, Fasciclin domain, Gene regulation

## Abstract

Expression of *fdp,* encoding a fasciclin I domain protein important for adherence in the hydrogen-producing bacterium *Rhodobacter sphaeroides,* was investigated under a range of conditions to gain insights into optimization of adherence for immobilization strategies suitable for H_2_ production. The *fdp* promoter was linked to a *lacZ* reporter and expressed in wild type and in PRRB and PRRA mutant strains of the Prr regulatory pathway. Expression was significantly negatively regulated by Prr under all conditions of aerobiosis tested including anaerobic conditions (required for H_2_ production), and aerobically regardless of growth phase, growth medium complexity or composition, carbon source, heat and cold shock and dark/light conditions. Negative *fdp* regulation by Prr was reflected in cellular levels of translated Fdp protein. Since Prr is required directly for nitrogenase expression, we propose optimization of Fdp-based adherence in *R. sphaeroides* for immobilized biohydrogen production by inactivation of the PrrA binding site(s) upstream of *fdp*.

## Highlights

• Adherence and immobilization of hydrogen-producing microbes improves H_2_ yields.
• *fdp* encodes an adherence factor in hydrogen-producer *Rhodobacter sphaeroides*.
• Expression of *fdp* is negatively regulated by the global Prr regulatory pathway.
• Negative regulation by Prr was demonstrated under all growth conditions tested.
• Fdp-based adherence may be optimised by inactivating the PrrA binding site(s) of *fdp.*

## Introduction

*Rhodobacter sphaeroides* belongs to the purple non-sulphur (PNS) group of bacteria that are widely recognized as potential ‘green energy’ producers of biohydrogen from solid food waste and food processing wastewater (reviewed in Refs. [[1], [2], [3], [4]]). There are several other bacterial groups that generate hydrogen such as the bio-photolytic microalgae and cyanobacteria [5,6], and some acidogenic thermophiles and mesophiles that perform dark fermentative hydrogen production [[7], [8], [9]]. Examples recently reported include hydrogen-producing clostridial strains isolated from landfill leachate sludge [10], that produce high yields of up to 4.7 mol H_2_/mol glucose [11]; *Clostridium sartagoforme* and *Enterobacter cloacae* strains isolated from Sago industrial effluent [12]; extreme halophiles that produce biohydrogen from lignocellulose biomass in nearly saturated salt [13]; *Bacillus* spp. isolated from banana waste [14] and improved hydrogen production by bioaugmentation with thermophiles exampled by *Thermoanaerobacterium thermosaccharolyticum* used to enhance thermophilic hydrogen production from corn stover hydrolysate [15]. The use of consortia of these groups of microorganisms, derived either as endogenous species isolated from biomass or from other environmental sources and used to augment the natural microbial flora has also proved a successful strategy [[16], [17], [18], [19], [20]]. However, the photofermentative processes involved in hydrogen production performed by the PNS group (represented by *R. sphaeroides* but also including *Rhodopseudomonas capsulatus*, *R. palustris* and *Rhodospirillum rubrum*), has attracted more attention because of the higher conversion efficiency and yields expected from the conversion of substrate to hydrogen and the abilities to utilise food industry wastes and solar light energy of wide ranging wavelengths (522–860 nm) [1,3,21].

*R. sphaeroides* has attracted particular attention, not least because of its remarkable metabolic versatility; it is able to grow photoheterotrophically, photoautotrophically, fermentatively and using aerobic or anaerobic respiration [[21], [22], [23], [24]]. Photofermentation by PNS bacteria such as *R. sphaeroides* involves fermentation of organic substrates in the presence of light. Light results in the production and activity of a photosynthetic apparatus which facilitates electron flow from substrate to the [Mo–Fe]-nitrogenase. Nitrogenase activity results not only in fixation of nitrogen (in an irreversible reaction requiring large amounts of ATP via the F_0_F_1_-ATPase), but also conversion of H^+^ to hydrogen gas. *R. sphaeroides* also has a [Ni–Fe]-Hyd uptake hydrogenase enzyme which catalyses H_2_ oxidation in the presence of hydrogen gas. Although this enzyme also produces hydrogen under nitrogen excess conditions it is the nitrogenase that is considered to be the most important source of hydrogen generation [4]. Thus, hydrogen production in *R. sphaeroides* and other PNS bacteria provides a substrate for hydrogen oxidation reactions for energy generation and facilitates the activities of nitrogenase which catalyses the fixation of atmospheric nitrogen into a cellular source of reduced nitrogen [1,2].

There are a number of external factors reported to influence hydrogen production by *R. sphaeroides*, including culture medium composition (including nitrogen source and concentration, choice of organic substrate, use of mixed carbon sources and incorporation of certain metal ions), reducing agents, pH, light-dark period, illumination intensity, temperature, aerobiosis conditions and even low-intensity electromagnetic fields (e.g. Refs. [25,26] and reviewed in Refs. [2,4,9]). *R. sphaeroides* has been successfully used for biohydrogen production from biomass; e.g. it has recently been trialled for single-stage hydrogen production from hydrolyzed straw [27] and sugar beet molasses [28], and recently a new strain was identified for producing hydrogen using oil palm waste hydrolysate [29]. It has also been successfully used in co-culture with *Enterobacter aerogenes* for hydrogen production using *Calophyllum inophyllum* oil cake as complex carbon source [30]. Therefore, much is known about the external conditions needed to obtain and increase hydrogen production, though not all the mechanisms by which they work are yet understood.

**Fig. 1:**
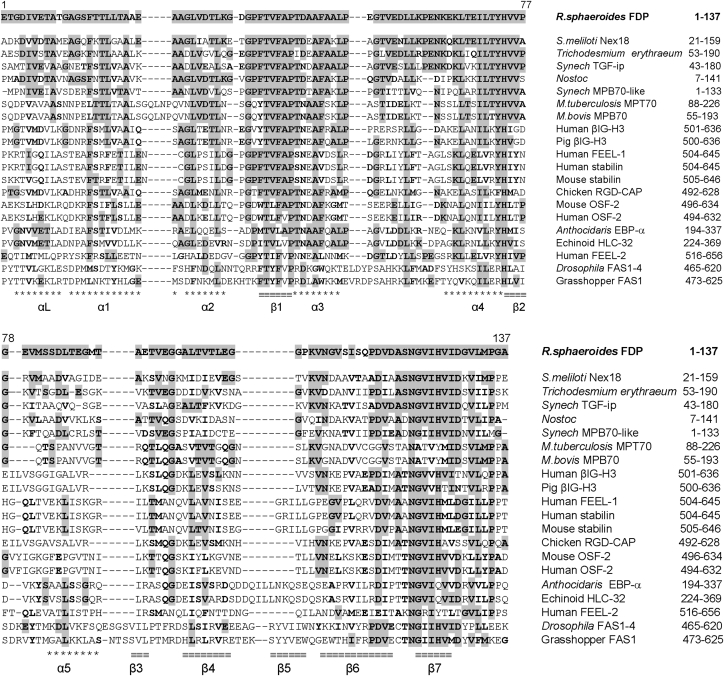
Alignment of *R. sphaeroides* Fdp with fasciclin-1 proteins. Amino acid residues that are identical to Fdp are in bold and grey shading; similar amino acid residues are in bold. *Sinorhizobium meliloti* Nex18, symbiotically-induced conserved protein Nex18 of *Sinorhizobium meliloti* (pir|F95334) 60/74% identical/similar; *Trichodesmium erythraeum* hypothetical protein (gb|ZP00071101.1) 60/72%; *Synech* TGF-ip, *Synechocystis* transforming growth factor-induced protein (pir|S76811) 56/73%; *Nostoc*, hypothetical protein all 4894 of *Nostoc* sp. PCC7120 (pir|AF2417) 57/74%; *Synech* MPB70-like, *Synechocystis* secreted protein MPB70-like slll735 (pir|S77329) 47/63%; *M. tuberculosis* MPT70, *Mycobacterium tuberculosis* major secreted MPT70 protein (gb|AAF13402.1/AF189006) 39/55%; *M. bovis* MPB70, *Mycobacterium bovis* major secreted protein MPB70 precursor (pir|A37195) 39/55%; Human βIG-H3, human transforming growth factor β-induced protein BIG-H3 (pir|I52996) 36/58%; Pig βIG-H3, pig transforming growth factor β-induced protein (kerato epithelin)(RGD-CAP)(sp|O11780|BGH3 PIG) 36/58%; Human FEEL-1, human FEEL-1 protein (dbj|BAC15606.1) 35/52%; Human stabilin, human stabilin 1 protein (emb|CAB61827.1) 35/52%; Mouse stabilin, mouse stabilin 1 protein (gb|AAL91671.2/AF2909141) 34/53%; Chicken RGD-CAP, RGD-containing collagen-associated protein (βIG-H3)(kerato epithelin) (dbj|BAA21479.1) 33/59%; Mouse OSF-2, mouse osteoblast-specific factor 2 (pir|S36109) 34/52%; Human OSF-2, human osteoblast-specific factor 2 (pir|S36110) 32/52%; *Anthocidaris* EBP-α, *Anthocidaris crassispina* EBP-α protein (dbj|BAA82956.1) 31/53%; Echinoid HLC-32, Echinoidea HLC-32 protein (gb|AAB32327.1) 30/50%; Human FEEL-2, human FEEL-2 protein (dbj|BAC15608.1) 29/49%; *Drosophila* FAS1-4, *Drosophila melanogaster* FAS1 4th fasciclin domain (pir|B29900) 29/52%; Grasshopper FAS1, grasshopper FAS1 (pir|A29900) 24/44%. Secondary structural data is derived from the structure of domain pair 3 and 4 of *Drosophila* FAS1 and is shown below the alignment (α-helix: ∗∗∗∗∗; β-strand: ≡≡≡≡). The conserved HI and H2 regions identified as protein interaction sites in several fasciclin I proteins are shown (residues 37-46 and 124-133 in Fdp, respectively).

Immobilization of PNS bacteria through biofilm formation has also been reported to be beneficial for hydrogen yields and opens up the possibilities of semi- or full-continuous culture methods for hydrogen production [1,[31], [32], [33]], including biophotoreactor technologies with enlarged surface areas [[34], [35], [36]]. Biofilm formation and adherence properties in *R. sphaeroides* are multifactorial, affected by flagellar location and number [37,38], chemotaxis [39], membrane cardiolipin [40], presence of functional fasciclin-1 domain protein (Fdp) [41], as well as by light-driven and other regulatory factors [42,43]. In the case of *R. sphaeroides* Fdp, insertionally-inactivated *fdp* knockout strains were reported to reduce cell adherence by 100-fold (in terms of cell number) [41]. Fdp resembles the fasciclin I (FAS1) domains found in proteins of higher organisms that have important roles in cell adhesion (Fig. 1). It also shares 60% identity (74% similarity) with the nodule-expressed Nex18 protein of *Sinorhizobium meliloti* [44], though there appear to be no homologues in other PNS bacteria (Fig. 1). The precise mechanism by which Fdp promotes cell adherence (a prerequisite for biofilms) in *R. sphaeroides* remains unknown [41]. Clearly, a deeper understanding of the factors important for establishment and maintenance of *R. sphaeroides* in an immobilized state will be important for improved hydrogen yields reportedly gained through immobilization, not least through employment of continuous flow photobioreactors which optimize microbial exposure to light and fresh nutrients and biomass substrates [e.g. 35]. The aim therefore of the present study was to identify conditions for Fdp expression in *R. sphaeroides* that promote immobilization and which can therefore ultimately be applied to hydrogen production via nitrogenase. This was investigated by testing a range of growth, chemical and physical conditions on transcriptional expression of *fdp*, including anaerobic conditions with reduced NH_4_^+^. We show that *fdp* transcription is strongly repressed by the Prr global regulatory system in wild type *R. sphaeroides* under all laboratory conditions tested here. This leads us to propose the future development of a new strain mutagenesis strategy for optimizing hydrogen generation based on increased attachment and biofilm development mediated by Fdp in *R. sphaeroides* for use in bioreactors designed for continuous biohydrogen production, through promoter engineering upstream of the *fdp* gene that reduces or abolishes Prr repressor binding upstream of the *fdp* locus.

## Materials and methods

### Chemicals

The chemicals used in this study were purchased from Merck (Gillingham, Dorset, UK), VWR (Lutterworth, Leicestershire, UK), or Fisher Scientific (Loughborough, Leicestershire, UK) unless otherwise stated, and were of molecular biology grade.

### Bacterial strains, plasmids and growth conditions

All strains and plasmids are described in Table 1 [[45], [46], [47], [48], [49]]. *E. coli* strains DH5α, S17-1 and BL21[DE3] have been described previously and were routinely cultured aerobically in Luria-Bertani (LB) media by vigorous aeration of culture vessels, or on LB agar, at 37 °C as described in Ref. [50]. Where appropriate, media were supplemented with 50 μg mL^−1^ ampicillin and/or 50 μg mL^−1^ kanamycin or 500 μg mL^−1^ carbenicillin. Reporter plasmid transfer into *R. sphaeroides* was by conjugative transfer from *E. coli* S17-1 [45].

**Table 1:** Strains and plasmids used in this study.

Strain/plasmid	Relevant genotype/characteristics	Source/Reference
Strains		
*R. sphaeroides*		
NCIB 8253	Wild type	C.N. Hunter/[45]
PRRA	Derivative of NCIB 8253 wild type, Tn5 insertion in *prrA*; Km^R^	This work
PRRB	Derivative of NCIB 8253 wild type, Tn5 insertion in *prrB*; Km^R^	This work
FDP	Derivative of NCIB 8253 wild type, *kan* insertion in *fdp*; Km^R^	Eun-Lee Jeong/[41]
*E. coli*		
DH5-alpha	*supE44 δlacU169* ϕ80 *lacZ*δM15) *hsdRl7 recA1 endA1 gyrA96 thi-1 relA1*	[46]
S17-1	Mobilisation host. RP4-2 (Tc::Mu, Km::Tn7) *integrated into the chromosome: thi, pro, hsdR, hsdM*^*+*^*, recA, Tp*^*R*^*, Sm*^*R*^ Tra^*+*^	C.N. Hunter/[47]
BL21[DE3]	F^−^*ompT hsdS*_*B*_*(r*_*B*_^*-*^*m*_*B*_^*-*^*) gal dcm* (DE3)	Novagen
Plasmids		
pSUP202	Ap^R^ Tc^R^ Cm^R^; Mob^+^ Tra^−^ ColE1 replicon	C.N. Hunter/[47]
pSDP1	Tc^R^; Derivative of pRK415 with promoter-less *lacZ*. Replicates in *R. sphaeroides* and *E. coli*	[48]
pSDP-FDPP	Fdp reporter; pSDP1 with *fdp* promoter region inserted upstream of *lacZ*	This work
pREG464	Ap^R^, contains a 12 kb fragment of the *R. sphaeroides prr* cluster in pSUP202; *prrA*^+^*prrB*^+^*prrC*^+^	C.N. Hunter/[49]
pUX-Km	Ap^R^, Km^R^; pUC12 with Km^R^ gene flanked by symmetrical pUC12 multiple cloning sites	C.N. Hunter
pBluescript -SK	Ap^R^; pUC19 derivative. ColE1 *ori;* cloning vector with blue-white selection	Invitrogen
pET14b	Ap^R^; *E. coli* expression vector	Novagen

C.N.Hunter (University of Sheffield). Km^R^, kanamycin resistance; Ap^R^, ampicillin resistance; Tc^R^, tetracycline resistance; Tp^R^, trimethoprim resistance; Sm^R^, streptomycin resistance.

*R. sphaeroides* NCIB 8253 was cultured at 34 °C in M22 medium [45]; the *fdp* and *prrA* mutant strains were cultured in M22 containing 20 μg mL^−1^ kanamycin. Liquid M22 lacked added caesamino acids and contained 1.5 mM NH_4_^+^ which permits some nitrogenase expression under anaerobic conditions [51] but little/no hydrogen evolution anaerobically due to the absence of light and the presence of dissolved N_2_. Growth was measured using culture absorbance at 680 nm (A_680_). Aerobic growth of *R. sphaeroides* was achieved using vigorous shaking of 10 mL medium in 250 mL vessels or 500 mL in 2 L vessels. Semi-aerobic growth at 34 °C was carried out using 70 mL medium in 250 mL vessels, whilst anaerobic growth at 34 °C in the dark was achieved using M22 medium containing 60 mM dimethyl sulphoxide (DMSO).

*R. sphaeroides prrA* and *prrB* knockout mutants (PRRA and PRRB respectively) were constructed by transposon Tn5 mutagenesis of pREG464 [52,53]. Insertion sites in *prrA* or *prrB* were verified by restriction analysis and DNA sequencing. Kanamycin-resistant transconjugants were screened for loss of the suicide plasmid by Southern hybridization using parental pSUP202 as labeled probe. The correct location of the inserted transposon (and loss of intact *prrA* or *prrB* gene from the chromosome) was determined by restriction and Southern hybridization analysis. The phenotypes of the resulting strains were identical to those reported for these mutations previously [54,55], including photosynthesis- and nitrogenase-minus phenotypes, and were successfully complemented using a 4.8-kb *Bam*HI *prr (reg)* fragment described in Ref. [49].

Plasmids pBluescript-SK and pET14b have been described previously [53]. Plasmid pSUP202 is a *R. sphaeroides* suicide plasmid used in *fdp* and *prr* mutant construction and is the host plasmid for the *R. sphaeroides* genomic library and has been described previously [47]. The *R. sphaeroides* replicative pSDP1 reporter plasmid possessing a promoter-less *lacZ* gene, and pUX-Km, have both been described previously [48].

Isolation of the *fdp* gene has been described previously [56]. Construction of an insertionally-inactivated *fdp* mutant was described by Ref. [41].

### Reporter studies of *fdp* expression

The 592 bp promoter region of the *fdp* gene (−613 to −21 relative to the ATG start codon) was amplified by polymerase chain reaction (PCR) using pSUP202*fdp*-13 as template, and primers NIT1: 5′- ATGAGGTACCTCGAGGAGGGTCCGCAGCTCG -3′ and NIT2: 5′- ATGATCTAGATCGTGCCGTGCTTGGCCTGC -3′ (*Kpn*I and *Xba*I sites are underlined). The PCR fragment was purified and cloned into *Sma*I-cut pBluescript-SK to give pBSFDPP-2. The promoter insert was checked by sequencing, prior to digestion with *Kpn*I and *Xba*I, and ligation into *Kpn*I*-* and *Xba*I-digested pSDP1, a promoter-less *lacZ* reporter plasmid described previously [48]. The resulting plasmid, pSDP-FDPP, was checked by restriction analysis. pSDP-FDPP and control pSDP1 were introduced into *R. sphaeroides* wild type and PRR mutant strains by conjugation via *E. coli* S17-1. Transconjugants were selected in culture media containing 1 μg mL^−1^ tetracycline (plus 20 μg mL^−1^ kanamycin for the PRR strains). β-galactosidase reporter assays were performed as described [48]. Protein content was measured either using the Bio-Rad *DC* Protein Assay or by the method in Ref. [57]. Bovine serum albumin (Sigma-Aldrich, Poole, UK) was used as the calibrant.

### Separation of cell proteins by two-dimensional SDS-PAGE and identification of Fdp

Protein extracts of semi-aerobically grown *R. sphaeroides* were prepared by batch culture to mid-exponential phase (A_680_ of 0.6). Cells from 300 ml cultures were harvested by centrifugation at 4 °C and resuspended in 10 mL TGEND buffer (comprising 10 mM Tris.HCl pH 8.0, 10% (v/v) glycerol, 0.1 mM EDTA, 50 mM NaCl, 0.1 mM dithiothreitol (DTT), 500 μM phenylmethyl sulfonyl fluoride (PMSF) and 50 μM *N*-tosyl-l-phenylalanine chloromethyl ketone (TPACK); final pH 8.3) at 4 °C. Cell suspensions were sonicated on ice (4 × 15 s bursts with 45 s intervals on ice). Unbroken cells and cell debris were removed by centrifugation for 20 min at 29,000 g at 4 °C and the protein supernatents (soluble, cytoplasm plus periplasm) stored at −70 °C.

40 μg protein were diluted in rehydration buffer (8 M urea, 2% Triton X-100, Pharmalyte pH3-10, Amphiline pH 6–8 1.5%, DTT 100 mM, bromophenol blue trace) and applied to 11 cm Immobiline DryStrips (Pharmacia Biotech Inc, USA) with an immobilized pH nonlinear gradient, pH 3 to 10. The first dimension was performed on an IGP isoelectric focusing unit (Pharmacia Biotech Inc, USA), and the second dimension was performed in 8–18% polyacrylamide gels. Gels were stained either with Coomassie brilliant blue or by the modified silver staining method of [58]. For sequence determinations of native Fdp, proteins were blotted onto membrane, visualised with Coomassie Brilliant Blue stain, excised from the membrane and the N-terminal sequence determined by Edman degradation.

### Overexpression and purification of His_6_-tagged Fdp in *E. coli* BL21[DE3]

To overexpress Fdp, the *fdp* region 57 to 470 (relative to ATG, where A is position 1), which lacks the region encoding the signal peptide region (residues 1–18), was amplified by polymerase chain reaction using upstream primer SGINT1: 5′- TCAGCCATATGGAAACCGGAGACATCGTGGA -3′ (*Nde*I cloning site underlined), and downstream primer SGEL2: 5′- GCTAGGATCCGCATCAGGCGCCCGGCATCAGCACG -3′ (*Bam*HI site underlined), using pSUP202*fdp*-13 as template. The 470-bp fragment was purified by gel extraction and cloned into *Sma*I-digested pBluescript-SK to give pBlFDP470. The presence of inserts with correct sequence was verified by restriction digest analysis and sequencing. Plasmid pBlFDP470 was digested with *Bam*HI and *Nde*I, and the *fdp* fragment cloned into pET14b (Novagen® Merck Group, UK). The final expression construct, pET*fdp*470, expresses a Fdp protein with a N-terminal MGSS(H)_6_SSGLVPRGSHM sequence followed by Fdp starting at E-19. Verification of the N-terminal sequence of recombinant purified Fdp was performed by Edman degradation: approximately 3 μg of purified his-tagged Fdp was loaded onto 15% polyacrylamide resolving gels, and transferred to Fluorotrans™ membrane (Pall BioSupport, UK) by electroblotting for 1 h at 100 V using a Bio-Rad Mini Trans-Blot Cell. The proteins were visualised with Coomassie Brilliant Blue, excised from the membrane and the *N*-terminal sequence determined.

The construct was transformed into *E. coli* BL21 [DE3]; overexpression and purification were performed as described for RegA (PrrA) in Ref. [53].

### Western blotting

To verify the presence of recombinant His-tagged Fdp purified from IPTG-induced *E. coli* BL21[DE3]/pET14*fdp,* Western blotting was undertaken using an antibody that recognises the His_6_ motif as described previously [59]. Briefly, purified His_6_-Fdp (4 μg) was loaded on 15% SDS-polyacrylamide resolving gels. Following electrophoresis by standard methods [50], proteins were transferred to nitrocellulose membrane (Amersham Hybond-C) by electroblotting for 1 h at 100 V using a Bio-Rad Mini Trans-Blot Cell. The transfer buffer contained 25 mM Tris.HCl pH 8.3, 192 mM glycine, 20% methanol, 0.025% sodium dodecyl sulphate (SDS). Membranes were washed twice for 10 min with TBS buffer (10 mM Tris.HCl pH 7.5, 150 mM NaCl) at room temperature, and incubated for 16 h in 3% (w/v) bovine serum albumin in TBS buffer. Membranes were then washed twice for 10 min each time in TBSTT buffer (TBS buffer containing 0.05% (v/v) Tween-20, 0.2% (v/v) Triton X-100), and then once for 10 min in TBS buffer. A 1:1000 dilution of mouse anti-RGS(H)_6_ monoclonal antibody (Qiagen Ltd, Manchester, UK) was then prepared in TBS containing 3% BSA into which membranes were immersed for 1 h at room temperature. Following two washes for 10 min each time in TBSTT buffer and one wash for 10 min in TBS at room temperature, a 1:5000 dilution of goat anti-mouse IgG horse radish peroxidase conjugate (Stratech Scientific Ltd, Ely, UK) in TBS containing 10% (w/v) skimmed milk powder was added and the membranes incubated for 1 h at room temperature. Following four washes for 10 min each in TBSTT buffer, membranes were incubated with ECL Western blotting detection reagent (GE Healthcare, USA) and developed by autoradiography with Xograph film (Kodak Co., Herts, UK).

### Mass determinations using electrospray mass spectrometry

Samples of purified recombinant Fdp were prepared for electrospray mass spectroscopy by the method of [60] and analysed on a single quadrupole, bench top mass spectrometer (Platform II, Micromass UK Ltd). as described by Ref. [53]. Samples were dissolved in formic acid:methanol:water (1:1:1, v/v/v) and infused into the ionisation source at a flow rate of 10 μL per minute. Data were acquired over the appropriate *m/z* range and were processed using the MassLynx software supplied with the instrument. The *m/z* spectrum was transposed onto a true molecular mass scale for more facile identification using Maximum Entropy processing techniques. An external calibration is applied, using horse heart myoglobin (MW 16,951.49 Da) as the calibrant.

### Protein determinations

Protein content was measured using the Bio-rad DC Protein Assay Kit II (Bio-rad Laboratories Inc., Watford, Herts., UK) as outlined by the manufacturer, using bovine serum albumin as the standard.

## Results

### Confirmation of Fdp as a member of the fasciclin I protein superfamily

The open reading frame encoding Fdp (ORF RSP1409) was first identified as a FAS1 fasciclin I–like protein in Ref. [56] (Beta-Ig-H3/Fasciclin; https://www.uniprot.org/uniprot/Q3IXZ6). Fdp has a predicted signal peptide at the N-terminus (residues 1–18: (RKTLLALSLGLLAAPAFA)) suggesting a protein that is translocated across the inner membrane resulting in a mature 137-residue protein possessing the N-terminal sequence ETGDIVETATGA. By PSI-BLAST, the closest sequence similarity (60% identical; 74% similar) is to *Sinorhizobium meliloti* Nex18 (Fig. 1). It is also related (32–39% identity; 52–59% similarity) to *Mycobacterium tuberculosis* MPT70 and *M. bovis* MPB70 major secreted proteins [61], the fasciclin I domains of mammalian transforming growth factor β-induced proteins (βIG-H3 or RGD-CAP adhesion proteins, as indicated in UniProt) [62], and human osteoblast-specific factor 2 (OSF-2 or periostin) [63], which is thought to be involved in bone adhesion and is a ligand for αvβ5 integrin [64] (Fig. 1). *Drosophila* FAS1 domain 4 [65,66], which is responsible for axon guidance, has 29% identity to Fdp (Fig. 1). The common feature in all these proteins, where a function is known, is their involvement in protein-protein associations. The sequence similarities are quite striking, since fasciclin I domains generally exhibit low overall sequence conservation (<20%) [66]. The two regions of high conservation recognized for the FAS1 superfamily (H1 and H2) are also strongly conserved in this putative protein. Taken together with the NMR structure of Fdp described previously [41,56], it is clear that this protein is a member of the fasciclin I protein superfamily. One unusual aspect of this particular fasciclin-domain protein is that it occurs in a free-living bacterium, and fortuitously this free-living species is well characterized regarding its physiology, metabolic versatility, molecular bases for responses to environmental change and it is also amenable to knock-out strategies. Indeed the role of Fdp in cell adherence properties of *R. sphaeroides* has already been established; Fdp appears to promote cell adherence as shown by insertional activation studies in which inactivation of *fdp* resulted in a 100-fold reduction in numbers of adherent cells in a *R. sphaeroides* adherence assay [41]. Here we investigate the regulation of expression of this adherence factor in *R. sphaeroides*, which could yield important knowledge for the establishment and continuous immobilization of bacterial cells in bioreactors.

### Transcription of Fdp is negatively regulated by the Prr signaling pathway under anaerobic and other growth conditions

Prr is a major regulator that senses changes in external redox potential and serves as a global switch in gene expression for many genes in *R. sphaeroides* [[67], [68], [69]]. To investigate whether this global environment-responsive regulator controls *fdp* transcription, reporter studies were undertaken using the promoter region of the *fdp* gene linked to a *lacZ* reporter gene, which was expressed in both wild type and PRR mutants. Table 2 shows activity of the *fdp* promoter under different aerobiosis conditions, as shown by β-galactosidase measurements of *R. sphaeroides* extracts from stationary-phase cells harbouring pSDP-FDPP, a pRK415-based replicative reporter plasmid carrying 592-bp of *fdp* upstream sequence transcriptionally linked to *lacZ*. Experiments were carried out using wild type, plus two mutants PRRA and PRRB in which the *prrA* (encoding the response regulator PrrA) and *prrB* (encoding the redox sensor kinase PrrB) genes, respectively, were insertionally inactivated. Anaerobic conditions were achieved using dark conditions in the presence of DMSO rather than light conditions for light harvesting, since PRR mutants are unable to grow photosynthetically. Table 2 shows that levels of *fdp* expression levels in aerobic and semi-aerobic cells of wild type grown on succinate-lactate medium were similar (ΔA_405_ units/min/mg protein = 93–100 × 10^3^), but were slightly lower under anaerobic conditions (required for nitrogenase expression and thereby hydrogen generation) [1] (and under which the Prr pathway generates a higher level of phosphorylated PrrA, Prr-P) (ΔA_405_ units/min/mg protein = 64 × 10^3^) [70] (Table 2). Expression was significantly higher (3.7–40.3-fold) in both PRRA and PRRB strains compared with wild type under all conditions of aerobiosis in these cells grown on glucose or succinate-lactate (Table 2), demonstrating that the Prr system exerts negative control of *fdp* transcription under both aerobic and anaerobic nitrogenase-expressing conditions. Presumably sufficient transcriptionally-active PrrA or PrrA-P must occur for the efficient repression of the *fdp* promoter region observed under all aerobiosis conditions. The fold effect on expression levels in PRR mutants appears to be less marked under increasingly anaerobic conditions (though nonetheless significant), possibly suggesting that PrrA (which predominates under aerobic conditions compared with PrrA-P), is the overall repressor, and/or alternatively that additional aerobiosis-responsive regulators are regulating to different degrees under these conditions.

**Table 2:** Activity of the *fdp* promoter in stationary phase wild type, PRRB and PRRA strains cultured under different aerobiosis conditions.

Growth conditions	β-galactosidase (ΔA_405_/min/mg protein × 10^3^)							
	Wild type		PRRB			PRRA		
	Control	pSDP-FDPP	Control	pSDP-FDPP	Fold^a^	Control	pSDP-FDPP	Fold^a^
**M22 succinate/lactate**								
Aerobic	4.2 (3.1)	100.0 (19.2)	4.9 (4.3)	934.9 (121.8)	9.7	4.4 (7.4)	3487.5 (189.4)	36.4
Semi-aerobic	16.6 (5.8)	92.8 (10.1)	4.4 (1.6)	283.7 (15.5)	3.7	11.8 (1.9)	580.3 (14.4)	7.5
Anaerobic	16.5 (2.9)	64.4 (1.1)	6.3 (0.4)	252.8 (9.4)	5.2	10.1 (1.7)	279.3 (51)	5.6
**M22 glucose**								
Aerobic	6.1 (1.1)	83.9 (2.9)	5.4 (1.8)	1892.4 (18.2)	24.3	9.0 (0.6)	3140.7 (86.6)	40.3
Semi-aerobic	2.2 (0.9)	74.7 (3.4)	13.5 (0.9)	1098.6 (2.3)	15.0	4.6 (1.4)	2771.5 (80.9)	38.2
Anaerobic	4.4 (1.3)	67.7 (15.0)	2.0 (1.9)	507.8 (142.1)	6.8	3.6 (0.4)	370.0 (103.5)	5.0

The *fdp* promoter region was inserted upstream of *lacZ* as described in Methods, resulting in pSDP-FDPP. All growth experiments were conducted at 34 °C in the dark, and anaerobic growth was achieved by supplementing M22 medium with 60 mM dimethyl sulphoxide (DMSO). Growth (A_680_) was monitored and cells harvested in late stationary phase (aerobic cultures - 30–36 h; semi-aerobic cultures - 3 days; and anaerobic DMSO-grown cultures – 9 days). β-galactosidase measurements are means derived from four separate experiments, each set performed at least in triplicate (standard deviation values in parentheses). The enzyme levels produced in corresponding pSDP1-harboring control cells are shown. Bracketed values show the standard deviation values.

a Ratio of expression levels in PRR strains compared with wild type, calculated after subtraction of control pSDP1 activity.

Expression of *fdp* was less elevated in the PRRB strain compared with PRRA under aerobic and semi-aerobic growth conditions of aerobiosis, but levels were approximately equivalent in PRRA and PRRB strains under anaerobic conditions. This suggests that whilst there is a role for PrrB in *fdp* regulation under all aerobiosis conditions (shown by the elevated levels of reporter in the PRRB strain), under anaerobic conditions the loss of PrrB in PRRB exerts no greater or lesser effect on *fdp* transcription than loss of PrrA-P in PRRA, suggesting that PrrA-P derived only from PrrB acts as the repressor under anaerobic conditions in wild type cells and/or that any additional regulators present exert their effects equally on *fdp* expression in anaerobically-cultured PRRB and PRRA strains (Table 2).

A similar trend was observed using glucose-containing medium, though reporter levels (and fold effects) were overall higher in aerobic and semi-aerobic mutant cells compared with those grown in the same aerobiosis conditions using succinate-lactate medium (Table 2). Under anaerobic conditions on glucose (in common with succinate-lactate), *fdp* expression levels were elevated 5.0–6.8 fold in the absence of a functioning Prr pathway.

**Fig. 2:**
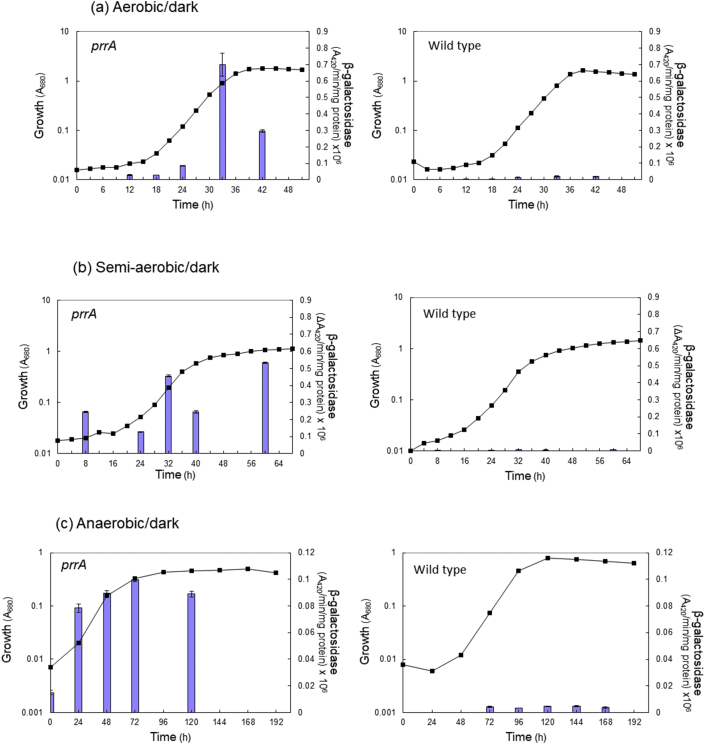
Activity of the *fdp* promoter in *R. sphaeroides* wild type and PRRA strains during aerobic, semi-aerobic and anaerobic batch growth. The *fdp* promoter region was inserted upstream of *lacZ* in reporter plasmid pSDP1 as described in Methods, resulting in pSDP-FDPP. A promoter-less control was also included throughout all growth experiments; reporter levels remained at the expected very low levels throughout these experiments. Plasmids were introduced into *R. sphaeroides* wild type and PRRA strains and maintained as described in Methods. Growth experiments were performed at 34 °C in M22 media under (a) aerobic/dark, (b) semi-aerobic/dark and (c) anaerobic/dark (in the presence of 60 mM DMSO) conditions for both wild type- and PRRA-transformed strains. Samples (1–50 ml) were taken for measurements of growth (absorbance at 680 nm, A680) (**−**■**−**) and duplicate β-galactosidase measurements (shown by the blue bars), as described in Methods. (For interpretation of the references to colour in this figure legend, the reader is referred to the Web version of this article.)

Reporter studies of cells harvested at different times during batch growth revealed that in the wild type strain, under all conditions of aerobiosis including anaerobic conditions, reporter levels remained at constant low levels throughout growth (Fig. 2). By contrast, reporter levels in anaerobic/dark-grown PRRA were significantly elevated, though once again relatively similar throughout growth. Under aerobic/dark conditions (and to a lesser extent under semi-aerobic/dark conditions), reporter levels appeared more variable and possibly growth-phase dependent in the PRRA mutant. Levels in the PRRA mutant increased during lag and early exponential phase under aerobic conditions and reached a maximum level in late-exponential phase, reaching up to 99-fold those of wild type cells in the same phase of growth (Fig. 2). This may suggest the presence of additional regulators governing *fdp* expression, in addition to Prr. Indeed, the higher *fdp* expression observed in late exponential phase cells cultivated under aerobic conditions is reminiscent of gene expression control governed by quorum-based systems [71]. To perform preliminary investigations on whether quorum sensing in *R. sphaeroides* [72] could possibly play a role in regulation of *fdp*, reporter studies were undertaken using early-exponential phase aerobically-grown cells from wild type and PRRA strains and to which were added sterile culture supernatants from stationary phase wild type cells (shown to accumulate 7,8-cis-*N*-(tetradecenoyl) homoserine lactone, [72]) to constitute 10% of the total culture volume. Expression levels of *fdp* were compared to those of untreated cells after 1 h further incubation. Addition of the culture supernatant did not significantly affect expression levels; expression levels in wild type were 0.6-fold compared with untreated cells whilst in the PRRA strain levels were only 1.3-fold those of the control (Table 3). Therefore, these preliminary experiments indicate that quorum sensing plays no detectable role in *fdp* regulation, but further investigations should be conducted to confirm this.

**Table 3:** Comparison of *fdp* promoter activity in wild type and PRRA strains under different growth conditions.

Treatment	Strain	β-galactosidase (ΔA_405_/min/mg protein × 10^3^)^a^							
		Control untreated cultures^c^				Addition of sterile supernatent^c^			Fold^*b*^
		Control		pSDP-FDPP		Control		pSDP-FDPP	
1	Wild type	1.5 (1.5)		24.4 (7.8)		6.5 (1.8)		21.4 (1.3)	0.6
	PRRA	11.7 (3.1)		760.0 (347.7)		12.3 (5.8)		977.0 (58.4)	1.3
				32.7 fold^b^				45.1 fold^b^	
		M22 succinate lactate^d^				Luria-Bertani medium^d^			Fold^*b*^
		Control		pSDP-FDPP		Control		pSDP-FDPP	
2	Wild type	6.1 (1.0)		52.5 (6.7)		24.5 (25.8)		165.9 (24.5)	3.0
	PRRA	4.3 (1.5)		5185.8 (297.7)		4.4 (0.8)		21806 (3667.8)	4.2
				111.7 fold^b^				154.2 fold^b^	
		Control conditions^e^		Heat shock^e^		Fold^b^	Cold shock^e^		Fold^b^
		Control	pSDP-FDPP	Control	pSDP-FDPP		Control	pSDP-FDPP	
3	Wild type	5.0 (5.5)	183.5 (5.6)	3.0 (3.3)	254.4 (18.2)	1.4	1.3 (0.3)	209.3 (20.9)	1.2
	PRRA	7.3 (3.7)	2770.6 (64.3)	9.8 (0.83)	7356.6 (225.6)	2.7	16.8 (14.8)	3868.6 (191.2)	1.4
			15.5 fold^b^		29.2 fold^b^			18.5 fold^b^	
		Dark^b,f^				Light^f,g^			Fold^b^
		Control		pSDP-FDPP		Control		pSDP-FDPP	
4	Wild type	3.2 (3.0)		141.4 (13.9)		5.5 (1.0)		178.2 (4.2)	1.3
	PRRA	2.2 (0.0)		1802.4 (55.4)		16.0 (8.9)		5457.0 (24.5)	3.0
				12.7 fold^b^				31.5 fold^b^	

^a^The *fdp* promoter region was inserted upstream of *lacZ* as described in Methods, resulting in pSDP-FDPP (Table 1). β-galactosidase measurements are means derived from triplicate measurements (standard deviation values in parentheses). The enzyme levels produced in corresponding pSDP1-harbouring control cells are shown.

^b^Fold is the ratio of expression levels in the presence and absence of treatment (or Prr mutant: wildtype levels), calculated after subtraction of control pSDP1 activity. See individual treatments for more specific detail.

**Treatment 1:** Activity in early exponential phase cells in response to addition of sterile supernatent from stationary phase cells. ^c^Aerobic dark growth at 34 °C (A_680_) was monitored and either sterile culture supernatent from stationary phase cells were added or no addition made (Control), to early log phase cells.

**Treatment 2:** Activity in stationary phase cells cultured aerobically in rich (LB) and minimal M22 succinate-lactate media. ^d^Aerobic dark growth at 34 °C (A_680_) was monitored (A_680_) and cells harvested in stationary phase (30–36 h). Fold: ratio of expression levels in LB medium compared with those in M22 medium, calculated after subtraction of control pSDP1 activity.

**Treatment 3:** Activity in stationary phase cells following exposure to temperature shock. ^e^cultures were grown aerobically in M22 succinate-lactate medium at 34 °C in the dark until early stationary phase before heat shock at 42 °C or cold shock at 5 °C for 4 h and cell harvesting. Fold: Ratio of expression levels compared with continued standard conditions, calculated after subtraction of control pSDP1 activity.

**Treatment 4:** Activity in stationary phase cells cultured in the light or dark. ^f^Aerobic growth in M22 succinate-lactate medium at 34 °C in the dark or light was monitored (A_680_) and cells harvested in stationary phase (30–36 h). Fold: ratio of expression levels in the light compared with those in the same medium in the dark under aerobic conditions at 34 °C, calculated after subtraction of control pSDP1 activity. ^g^86 W m^2^.

Although levels of *fdp* expression were consistently low in wild type (compared with PRR mutants) under all conditions of aerobiosis tested (Table 2), some variation in expression levels nonetheless occurred, specifically there are significantly lower levels of expression under anaerobic conditions on succinate/lactate medium compared with aerobic and semi-aerobic conditions in the same medium (Table 2). To investigate whether other environmental factors can also affect *fdp* transcription in wild type, the effects of complex versus defined medium, heat versus cold shock and light versus dark conditions were investigated. For comparative purposes, strains were all cultured under aerobic conditions, so that a wider range of conditions could be investigated at a practical level. Thus, the anaerobic conditions required for nitrogenase expression were not specifically investigated here. The study also included the effects of these factors on *fdp* expression in PRRA strain, to determine whether any variation also occurs in the absence of the Prr pathway. The results in Table 3 demonstrate that *fdp* expression was sensitive to growth medium composition in both wild type and PRRA; expression was upregulated 3-fold in the wild type and 4.2-fold in PRRA in Luria-Bertani complex medium compared with M22 succinate-lactate medium (Table 3). There was no significant effect of cold shock in both strains nor of heat shock or light compared with dark on wild type expression. Expression in PRRA was 2.7-fold elevated upon heat shock and 3-fold elevated under light conditions compared with identical dark conditions (Table 3). Thus, growth medium composition (complex versus defined) significantly affected expression in the wild type as well as in PRRA, though most changes in expression levels were observed in the PRRA mutant strain. It is difficult to draw conclusions about the nature of the mechanisms by which such different regulation occurs.

To investigate possible regulatory mechanisms by which *fdp* is regulated under different growth conditions, studies were focused on reporter studies using pyruvate-grown cells to ascertain whether the global signaling molecule acetyl phosphate which occurs in *R. sphaeroides* [73] may affect *fdp* expression. When pyruvate is the carbon source, levels of the small phospho donor acetyl phosphate are elevated [74,75]. Acetyl phosphate is a global signaling molecule that regulates many bacterial cellular processes including nitrogen assimilation, osmoregulation, flagellar biogenesis, pilus assembly, capsule biosynthesis, biofilm development, and pathogenicity [76]. One way it has been shown to exert its regulatory effects is by direct phosphorylation of response regulators of two-component systems, including *R. sphaeroides* response regulators [74,77,78]. In wild type and *fdp* mutant strains, pyruvate-grown cells consistently expressed significantly less *fdp* compared with succinate/lactate grown cells, possibly indicating greater repression by Prr, though effects due to additional regulators cannot be ruled out (Table 4). However, in the PRRA mutant strain which lacks the PrrA response regulator, the fold increase in expression levels was significantly higher (83-fold and 58-fold in semi-aerobic and anaerobic conditions, respectively) in pyruvate-grown cells compared with succinate/lactate-grown cells (10-fold) (Table 4), suggesting a possible role for acetyl phosphate and/or the presence of additional phosphorylatable regulators of *fdp* expression in addition to the Prr pathway under anaerobic (and semi-aerobic) conditions.

**Table 4:** Comparison of succinate-lactate and pyruvate carbon sources on the activity of the *fdp* promoter in wild type, FDP and PRRA strains.

Growth conditions	β-galactosidase (ΔA_405_/min/mg protein × 10^3^)				
	Wild type	FDP	Fold^a^	PRRA	Fold^a^
**Semi-aerobic**					
Succinate-lactate	51 (10)	48 (22)	0.9	517 (63)	10.1
Pyruvate	10 (0.6)	15 (2)	1.5	832 (37)	83.2
**Anaerobic**					
Succinate-Lactate	40 (5)	50 (60)	1.3	410 (20)	10.3
Pyruvate	10 (0.6)	30 (10)	3.0	580 (70)	58.0

The *fdp* promoter region was inserted upstream of *lacZ* as described in Methods, resulting in pSDP-FDPP. All growth experiments were conducted in M22 medium at 34 °C in the dark, and anaerobic growth was achieved by supplementing M22 medium with 60 mM dimethyl sulphoxide (DMSO). Growth (A_680_) was monitored and cells harvested in late exponential phase. β-galactosidase measurements are means derived from triplicate measurements (standard deviation values in parentheses).

a Ratio of expression levels compared with wild type, calculated after subtraction of control pSDP1 activity.

### Comparisons of Fdp protein levels in wild type and Prr mutants *in vivo*

As shown in all the reporter data described above, Fdp expression is significantly lower in the wild type strain compared with the PRR mutants. To determine whether these differences are also reflected *in vivo* with regard to the final translated Fdp protein, 2D SDS-PAGE analysis of cell extracts was undertaken. The Fdp protein possesses a putative signal peptide at the N-terminus, suggesting that the protein is secreted either externally or onto the cell surface, into the periplasm or is membrane–associated. Since post-translationally modified Fdp is of relatively low molecular mass (13.8 kDa), and is predicted to possess a low predicted pI (3.96) compared with other *R. sphaeroides* proteins, we reasoned that it should be possible to separate and identify the protein in two-dimensional SDS-PAGE of *R. sphaeroides* cell fractions.

**Fig. 3:**
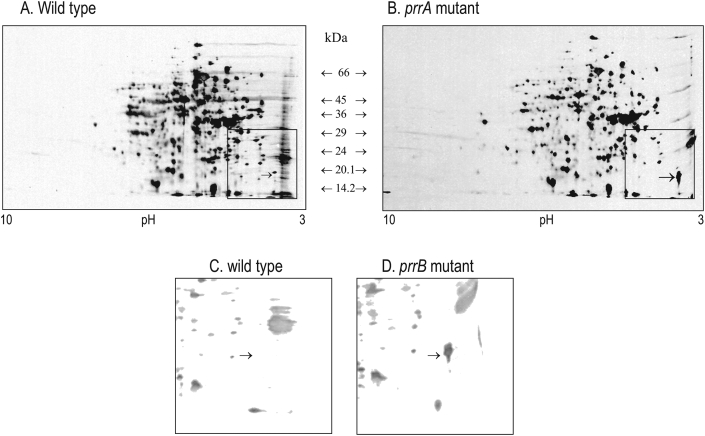
Separation of soluble proteins of wild type, PRRA and PRRB strains of *R. sphaeroides* by two-dimensional SDS-polyacrylamide gel electrophoresis. Extracts (40 μg protein) from mid-exponential phase cells cultured under semi-aerobic/dark conditions at 34 °C were separated as described in Methods. Proteins were extracted from: (A) wild type; (B) PRRA (an insertionally inactivated *prrA* mutant). The boxed area is the region showing the Fdp protein (indicated by an arrow). This boxed area is expanded for comparisons of: (C) wild type and (D) PRRB (an insertionally inactivated *prrB* mutant) extracts. Typical results from five comparisons of wild type versus PRRA, and one comparison of wild type versus PRRB.

**Fig. 4:**
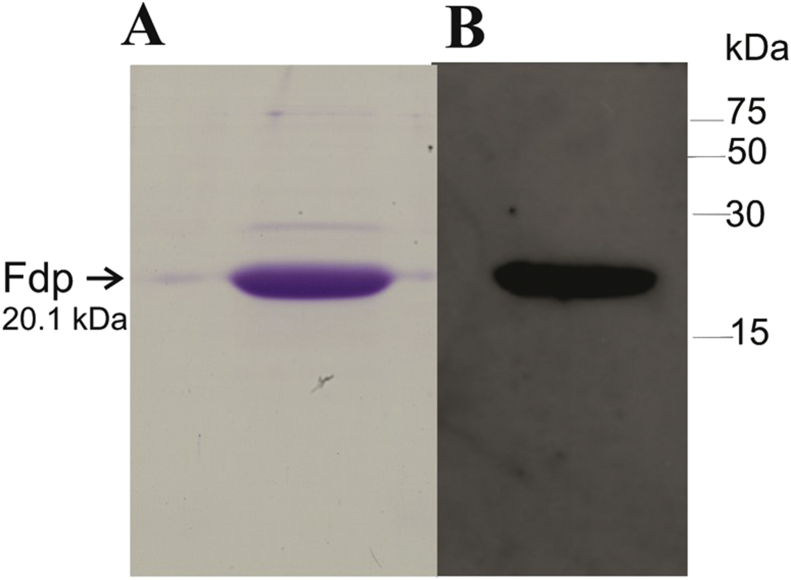
Production and verification of purified His-tagged Fdp. (A) SDS-polyacrylamide gel (6% stacking and 15% resolving gel) showing 4 μg purified recombinant Fdp (predicted molecular mass 16004.8 Da) with apparent molecular mass of 20,100 Da, visualised using Coomassie blue staining. (B) Western blot using 4 μg of purified Fdp with an INDIA His probe to detect the presence of the N-terminal hexahistidine tag. Arrow denotes the position of the Fdp protein. The positions of molecular mass markers are indicated. (For interpretation of the references to colour in this figure legend, the reader is referred to the Web version of this article.)

Soluble extracts (including periplasmic fractions) of washed semi-aerobic wild type and PRR mutant cells were used in the 2D SDS-PAGE analysis (Fig. 3). The correct protein spot and position in the gels were identified by N-terminal sequencing (sequenced as ETGDIVETATSA, compared to the Fdp protein in the *R. sphaeroides* genome database which is ETGDIVETATGA). Characteristically, it runs anomalously in the approximate technique of SDS-PAGE, with an apparent molecular mass of 17,300 Da, higher than the predicted 13,800 Da. This is a characteristic also observed using a purified his-tagged version of Fdp expressed in *E. coli*; His_6_-Fdp possesses an apparent molecular mass of 20,100 Da in SDS-PAGE (Fig. 4) but mass spectrometry reveals a mass of 16003.9 ± 1.6 Da, in good agreement with the expected theoretical value for the recombinant protein (16,004.8 Da).

Fig. 3 shows that in PRRA and PRRB soluble extracts, levels of Fdp are significant in comparison with other cellular soluble proteins, confirming that washed cells possess abundant levels of mature, post-translationally modified Fdp. Taken together with the N-terminal sequencing data, these results demonstrate that the predicted N-terminal signal peptide is indeed cleaved *in vivo*, and that mature Fdp is therefore presumably exported across the inner membrane and at least into the periplasm in these strains. Fdp levels were lower or barely detectable in the wild type strain (Fig. 3), a feature consistent with the findings described above for Fdp transcription.

## Discussion

The present study clearly demonstrates that expression of the *fdp* gene encoding a protein involved in adherence [41] is negatively regulated by the Prr global regulator in *R. sphaeroides*. Under a wide range of growth conditions tested here including different carbon sources, conditions of aerobiosis (including anaerobic conditions suitable for nitrogenase expression), rich versus defined growth media, heat/cold shock, and light versus dark conditions, elevated *fdp* promoter activity was consistently observed in PRRA and PRRB mutants compared with wild type, ranging from 3.7 to 154-fold (Table 2, Table 3, Table 4). Increased levels of promoter activity in PRR mutants were also observed throughout batch growth under all aerobiosis conditions (Fig. 2) and whilst the highest levels of promoter activity were seen in late exponential phase cells under aerobic/dark conditions, no evidence of quorum-based regulation was found (Table 3), though further study is needed to confirm this. Interestingly Prr repression occurred under all conditions of aerobiosis, suggesting either sufficient levels of PrrA-P under all these aerobiosis conditions for repression, or that unphosphorylated PrrA is also able to repress *fdp*. The regulatory activity of PrrA (or analogous RegA in other species) as well as of PrrA-P (RegA-P) has been documented previously [77,[79], [80], [81]].

In the absence of the Prr pathway, the increased levels of *fdp* expression varied depending on growth conditions, possibly suggesting the involvement of additional regulators involved in Fdp regulation. This was further supported by growth experiments using pyruvate as carbon source, in which elevated levels of the global signaling molecule acetyl phosphate are present; in PRR mutants growing on pyruvate, reporter levels were significantly elevated still further (Table 4), suggesting possible regulation by phosphorylatable control systems, such as two-component signal transduction systems. The effects on *fdp* expression measured using our reporter assay system were also reflected in the levels of translated Fdp protein observed in cell extracts *in vivo* (Fig. 3).

Evidence for the adherent properties of the wild type strain of *R. sphaeroides* used in this study has been reported previously [41]. The strain was shown here to exhibit low levels of *fdp* expression resulting in low, barely detectable levels of post-translationally modified Fdp protein in which the signal peptide has been removed *in vivo* (Table 2, Table 3, Table 4, Fig. 3). Such low levels are surprising, but presumably these levels in the wild type are nonetheless sufficient to support cell adherence. There were low but detectable levels of *fdp* expression in the wild type under all conditions tested, and yet there were some limited levels of variation in these expression levels under different conditions. For example, expression levels in wild type cells cultured anaerobically in M22 succinate/lactate medium were 64% of those measured aerobically in the same medium (Table 2). Similarly, *fdp* expression in wild type cells cultured anaerobically with pyruvate as carbon source was approximately 5-fold lower than in cells grown in the same medium semi-aerobically with succinate-lactate as carbon source (Table 4), and aerobic wild type cells cultured in rich LB medium exhibited 3-fold elevated levels of *fdp* expression over cells cultured in defined M22 medium (Table 3). These variations may be due to variable regulation by Prr itself as reported for the analogous Reg pathway in *R. capsulatus* which regulates in a variable way different gene sets depending on growth conditions [81]. Alternatively, other Prr-independent regulatory activity may be occurring. In light of the strong regulation exerted by Prr regulation in wild type demonstrated in this study by the significantly derepressed levels of *fdp* expression observed in the PRR mutants, the latter of these two possibilities appears to be the most likely explanation for the relatively low (but significant) levels of variation seen in the wild type.

The question therefore is why Fdp expression should be subject to such strong regulation by Prr and possibly other phosphorylatable regulators under most growth conditions as demonstrated here. One possible explanation is that there may be occasions in which it is advantageous for the bacterium to experience a full reversal or partial loss of adherence ability, for example in order to enter a motile phase. Perhaps there are particular environmental conditions which occur in nature (and which were not possible to replicate in the laboratory environment), that facilitate full repression of Fdp levels equivalent to a full shut down of Fdp in the wild type, resulting in motile phase non-adherent cells. A previous study established that, in terms of cell numbers, adherence is reduced approximately 100-fold in an insertionally-inactivated *fdp* mutant [41]. In this regard it is relevant to note that *Rhodobacter* mutants in the global Prr/Reg regulatory pathway, and shown here to exhibit significantly elevated levels of Fdp in *R. sphaeroides*, are defective in aerotaxis and motility [81,82]. It is not suggested here that there must therefore be a direct link between elevated Fdp levels and loss in motility and aerotaxis, as Prr is a global regulator affecting many processes, but rather that such characteristics of Prr mutants makes the above hypothesis difficult to test. Another difficulty with testing this possibility is that in the present study no laboratory conditions were identified in which Fdp levels were fully repressed in the wild type. Nonetheless, with regard to promoting permenant adherence and thereby immobilization in a bioreactor environment, we propose that engineering of the PrrA binding site upstream of the *fdp* promoter to inhibit binding by the PrrA/PrrA-P repressor may be a useful future strategy. Development of the Prr mutants themselves, which are already lacking PrrA/PrrA-P binding and produce desirably elevated levels of Fdp, are not suitable in this case as they are defective in expression of nitrogenase for H_2_ production and other key metabolic processes such as photosynthesis and CO_2_ fixation required under light anaerobic conditions [67,70,81]. Therefore, a mutagenesis strategy designed to specifically abolish or reduce PrrA binding in the *fdp* promoter region and thereby ensure either strongly elevated levels of Fdp, or levels that are moderately higher than wild type levels, may be a fruitful line of future investigation.

Progress has previously been made in improving hydrogen yields through mutant analysis and genetic/metabolic engineering strategies, mainly targeting the activities of the uptake hydrogenase, poly-3-hydroxybutyric acid synthesis, nitrogenase and light harvesting systems under defined external conditions [2,3,23,[83], [84], [85], [86], [87]]. Not many reports have yet appeared on mutations in transcriptional regulators; however, the studies of [2,87,88] investigated the effects of HupR, HupT, NifA and NifL mutations for improving hydrogen yield in PNS bacteria, with success in improving hydrogen production. Searches using the consensus sequence for PrrA DNA binding in *R. sphaeroides* [89,90] reveal two possible binding sites for PrrA to the *fdp* upstream region, both of which occur in the promoter fragment used in the reporter studies described above. One starts at position −432 (5′-GCGCCGGCATTCTGCGC). In common with sites of other repressed genes such as hydrogenase (*hup*), this site has a rather long half-site spacing [89]. The second possible site starts at −419 (5′-GCGCCGGATCGC) and possesses a relatively short 6-nt half-site spacing [89]. Following confirmation of these PrrA binding sites, a comprehensive mutagenesis programme can be initiated.

## Conclusions

Expression of the *fdp* gene, which encodes an adherence factor in *R. sphaeroides*, is negatively regulated by the global Prr regulatory pathway. Strains defective in either the sensor kinase PrrB or the response regulator PrrA of this pathway possess significantly elevated levels of *fdp* promoter activity, which is also reflected in the levels of translated Fdp protein in *R. sphaeroides* cells *in vivo*. One strategy to optimize or increase adherence properties of *R. sphaeroides* in immobilized bioreactor applications might be to generate altered strains in which the Prr repressor activity has been reduced or removed. Mutations in the *prrA* or *prrB* genes themselves is not feasible, as they are required for expression of nitrogenase. We therefore propose targeted mutagenesis of the PrrA binding site upstream of the *fdp* gene to reduce or remove binding by the PrrA repressor specifically at this site and thereby enhance expression of the *fdp* adherence factor.

## Declaration of competing interest

The authors declare that they have no known competing financial interests or personal relationships that could have appeared to influence the work reported in this paper.

## References

[1] Basak N., Kumar Jana A., Das D., Saikia D. (2014). Photofermentative molecular biohydrogen production by purple non-sulfur (PNS) bacteria in various modes: the present progress and future perspective. Int J Hydrogen Energy.

[2] Wang Y., Zhou P., Gao R. (2016). Advances in the genetic modification of *Rhodobacter sphaeroides* to improve hydrogen production. Renew Sustain Energy Rev.

[3] Ghosh S., Kulsoom Dairkee U., Chowdhury R., Bhattacharya P. (2017). Hydrogen from food processing wastes via photofermentation using purple non-sulfur bacteria (PNSB) – a review. Energy Convers Manag.

[4] Trchounian K., Sawers R.G., Trchounian A. (2017). Improving biohydrogen productivity by microbial dark- and photo-fermentations: novel data and future approaches. Renew Sustain Energy Rev.

[5] Oncel S., Sabankay M. (2012). Microalgal biohydrogen production considering light energy and mixing time as the two key features for scale-up. Bioresour Technol.

[6] Ananyev G.M., Skizim N.J., Dismukes G.C. (2012). Enhancing biological hydrogen production from cyanobacteria by removal of excreted products. J Biotechnol.

[7] Ghimire A., Frunzo I., Pirozzi F., Trably E., Escudie R., Lens P.N. (2015). A review on dark fermentative biohydrogen production from organic biomass: process parameters and use of by-products. Appl Energy.

[8] Ghosh S., Chowdhury R., Bhattacharya P. (2018). A review on single stage integrated dark-photo fermentative biohydrogen production: insight into salient strategies and scopes. Int J Hydrogen Energy.

[9] Sun Y., He J., Yang G., Sun G., Sage V. (2019). A review of the enhancement of bio-hydrogen generation by chemicals addition. Catalyst.

[10] Wong Y.M., Juan J.C., Ting A., Wu T.Y. (2014). High efficiency bio-hydrogen production from glucose revealed in an inoculum of heat-pretreated landfill leachate sludge. Energy.

[11] Wong Y.M., Wu T.Y., Ling T.C., Show P.L., Lee S.Y., Chang J.-S., Ibrahim S., Juan J.C. (2018). Evaluating new bio-hydrogen producers: *Clostridium perfringens* strain JJC, *Clostridium bifermentans* strain WYM and *Clostridium* sp strain Ade.TY. J Biosci Bioeng.

[12] Nizzy A.M., Kannan S., Anand S.B. (2020). Identification of hydrogen gas producing anaerobic bacteria isolated from sago industrial effluent. Curr Microbiol.

[13] Taroepratjeka D.A.H., Imai T., Chairattanamanokorn P. (2020). Biohydrogen production by extremely halophilic bacteria from the salt pan of Samut Sakhon, Thailand. Chiang Mai J Sci.

[14] Da Silva Mazareli R.C., Sakamoto I.K., Edson L. (2019). *Bacillus* sp isolated from banana waste and analysis of metabolic pathways in acidogenic systems in hydrogen production. J Environ Manag.

[15] Zhang K., Cao G.-L., Ren N.-Q. (2019). Bioaugmentation with *Thermoanaerobacterium thermosaccharolyticum* W16 to enhance thermophilic hydrogen production using corn stover hydrolysate. Int J Hydrogen Energy.

[16] Martinez-Burgos W.J., Sydney E.B., de Paula D.R. (2020). Biohydrogen production in cassava processing wastewater using microbial consortia: process optimization and kinetic analysis of the microbial community. Bioresour Technol.

[17] Weiser M., Thompson R., Cremonez P.A., Maniglia T.C. (2020). Production of biohydrogen by an anaerobic digestion process using the residual glycerol from biodiesel production as additive to cassava wastewater. J Clean Prod.

[18] Martinez-Burgos W.J., Sydney E.B., Brar S.K. (2019). The effect of hydrolysis and sterilization in biohydrogen production from cassava processing wastewater medium using anaerobic bacterial consortia. Int J Hydrogen Energy.

[19] Villa Montoya A.C., da Silva Mazareli R.C., Delforno T.P. (2020). Optimization of key factors affecting hydrogen production from coffee waste using factorial design and metagenomics analysis of the microbial community. Int J Hydrogen Energy.

[20] Rodrigues C.V., Santana K.O., Nespeca M.G. (2020). Energy valorization of crude glycerol and sanitary sewage in hydrogen generation by biological processes. Int J Hydrogen Energy.

[21] Zhang Q., Wang Y., Zhang Z., Lee D.-J., Zhou X., Jing Y., Ge X., Jiang D., Hu J., He C. (2017). Photo-fermentative hydrogen production from crop residue: a mini review. Bioresour Technol.

[22] Cogdell R.J., Fyfe P.K., Barrett S.J., Prince S.M., Freer A.A., Isaacs N.W., McGlynn P., Hunter C.N. (1996). The purple bacterial photosynthetic unit. Photosynth Res.

[23] Koku H., Eroğlu I., Gündüz U., Yücel M., Türker L. (2002). Aspects of the metabolism of hydrogen production by *Rhodobacter sphaeroides*. Int J Hydrogen Energy.

[24] Arai H., Roh J.H., Kaplan S. (2008). Transcriptome dynamics during the transition from anaerobic photosynthesis to aerobic respiration in *Rhodobacter sphaeroides* 2.4.1. J Bacteriol.

[25] Al-Mohammedawi H.H., Znad H., Eroglu E. (2018). Synergistic effects and optimization of photofermentative hydrogen production of *Rhodobacter sphaeroides* DSM158. Int J Hydrogen Energy.

[26] Garimella S., Vimal A., Merugu R., Kumar A. (2012). Experimental optimization of green hydrogen production from phototrophic bacteria *Rhodobacter sphaeroides*. Rec Innov Chem Eng.

[27] Wang X., Fang Y., Wang Y. (2017). Single-stage photo-fermentative hydrogen production from hydrolyzed straw biomass using *Rhodobacter sphaeroides*. Int J Hydrogen Energy.

[28] Kars G., Alparslan U. (2013). Valorization of sugar beet molasses for the production of biohydrogen and 5-aminolevulinic acid by *Rhodobacter sphaeroides* OU001 in a biorefinery concept. Int J Hydrogen Energy.

[29] Pattanamanee W., Choorit W., Deesan C. (2012). Photofermentative production of biohydrogen from oil palm waste hydrolysate. Int J Hydrogen Energy.

[30] Arumugam A., Sandhya M., Ponnusami V. (2014). Biohydrogen and polyhydroxyalkanoate co-production by *Enterobacter aerogenes* and *Rhodobacter sphaeroides* from *Calophyllum inophyllum* oil cake. Bioresour Technol.

[31] Liao Q., Wang Y.-J., Wang Y.-Z. (2010). Formation and hydrogen production of photosynthetic bacterial biofilm under various illumination conditions. Bioresour Technol.

[32] Zagrodnik R., Thiel M., Seifert K. (2013). Application of immobilized *Rhodobacter sphaeroides* bacteria in hydrogen generation process under semi-continuous conditions. Int J Hydrogen Energy.

[33] Liu W., Yuan L., Wei B. (2016). Study on improvement of continuous hydrogen production by photosynthetic biofilm in interior illuminant reactor. J Environ Biol.

[34] Lin C.-Y., Thi Mai-Linh N., Chu C.-Y. (2018). Fermentative biohydrogen production and its byproducts: a mini review of current technology developments. Renew Sustain Energy Rev.

[35] Wang Y., Tahir N., Cao W. (2019). Grid columnar flat panel photobioreactor with immobilized photosynthetic bacteria for continuous photofermentative hydrogen production. Bioresour Technol.

[36] Wen H.-Q., Xing D.-F., Xie G.-J. (2019). Enhanced photo-fermentative hydrogen production by synergistic effects of formed biofilm and added L-cysteine. Renew Energy.

[37] Wilkinson D.A., Chacko S.J., Venien-Bryan C. (2011). Regulation of flagellum number by FliA and FlgM and role in biofilm formation by *Rhodobacter sphaeroides*. J Bacteriol.

[38] Schuhmacher J.S., Thormann K.M., Bange G. (2015). How bacteria maintain location and number of flagella. FEMS Microbiol Rev.

[39] Kojadinovic M., Armitage J.P., Tindall M.J. (2013). Response kinetics in the complex chemotaxis signaling pathway of *Rhodobacter sphaeroides*. J Royal Soc Interfaces.

[40] Lin T.-Y., Santos T.M.A., Kontur W.S. (2015). A cardiolipin-deficient mutant of *Rhodobacter sphaeroides* has an altered cell shape and is impaired in biofilm formation. J Bacteriol.

[41] Moody R.G., Williamson M.P. (2013). Structure and function of a bacterial Fasciclin I domain protein elucidates function of related cell adhesion proteins such as TGFBIp and periostin. FEBS Open Bio.

[42] Gomelsky M., Hoff W. (2011). Light helps bacteria make important lifestyle decisions. Trends Microbiol.

[43] Masuda S. (2013). Light detection and signal transduction in the BLUF photoreceptors. Plant Cell Physiol.

[44] Oke V., Long S.R. (1999). Bacterial genes induced within the nodule during the *Rhizobium*-legume symbiosis. Mol Microbiol.

[45] Hunter C.N., Turner G. (1988). Transfer of genes coding for apoproteins of reaction centre. J Gen Microbiol.

[46] Hanahan D., Glover D.M. (1985). Techniques for transformation of *E. coli*.

[47] Simon R., Priefer U., Puhler A. (1983). A broad host range mobilisation system for in vivo genetic engineering: transposon mutagenesis in Gram negative bacteria. Biotechnol.

[48] Duggan P.S., Parker S.D., Phillips-Jones M.K. (2000). Characterisation of a *Rhodobacter sphaeroides* gene that encodes a product resembling *Escherichia coli* cytochrome b561 and *R. sphaeroides* cytochrome b562. FEMS Microbiol Lett.

[49] Phillips-Jones M.K., Hunter C.N. (1994). Cloning and nucleotide sequence of *regA*, a putative response regulator gene of *Rhodobacter sphaeroides*. FEMS Microbiol Lett.

[50] Sambrook J., Fritsch E.F., Maniatis T. (1989). Molecular cloning: a laboratory manual.

[51] Akkӧse S., Gündüz U., Yücel M., Eroglu I. (2009). Effects of ammonium ion, acetate and aerobic conditions on hydrogen production and expression levels of nitrogenase genes in *Rhodobacter sphaeroides* O.U.001. Int J Hydrogen Energy.

[52] Hunter C.N. (1988). Transposon Tn5 mutagenesis of genes encoding reaction centre and light harvesting LH1 polypeptides of *Rhodobacter sphaeroides*. J Gen Microbiol.

[53] Potter C.A., Ward A., Laguri C., Williamson M.P., Henderson P.J.F., Phillips-Jones M.K. (2002). Expression, purification and characterisation of full-length heterologously expressed histidine protein kinase RegB from *Rhodobacter sphaeroides*. J Mol Biol.

[54] Eraso J.M., Kaplan S. (1994). PrrA, a putative response regulator involved in oxygen regulation of photosynthesis gene expression in *Rhodobacter sphaeroides*. J Bacteriol.

[55] Eraso J.M., Kaplan S. (1995). Oxygen-insensitive synthesis of the photosynthetic membranes of *Rhodobacter sphaeroides*: a mutant histidine kinase. J Bacteriol.

[56] Moody R.G., Phillips-Jones M.K., Williamson M.P. (2007). NMR assignment of the *Rhodobacter sphaeroides* fasciclin-1 domain protein (Fdp). Biomol NMR Assign.

[57] Schaffner W., Weissmann C. (1973). A rapid, sensitive, and specific method for the determination of protein in dilute solution. Anal Biochem.

[58] Blum H., Beier H., Gross H.J. (1987). Improved silver staining of plant proteins, RNA and DNA in polyacrylamide gels. Electrophoresis.

[59] Ma P., Yuille H.M., Blessie V., Göhring N., Iglói Z., Nishiguchi K., Nakayama J., Henderson P.J.F., Phillips-Jones M.K. (2008). Expression, purification and activities of the entire family of intact membrane sensor kinases from *Enterococcus faecalis*. Mol Membr Biol.

[60] Hufnagel P., Schweiger U., Eckerskorn C., Oesterhelt D. (1996). Electrospray ionization mass spectrometry of genetically and chemically modified bacteriorhodopsins. Anal Biochem.

[61] Nagai S., Matsumoto J., Nagasuka T. (1981). Specific skin-reactive protein from culture filtrate of *Mycobacterium bovis* BCG. Infect Immun.

[62] Suzuki H., Amizuka N., Kii I., Kawano Y., Nozawa-Inoue K., Suzuki A., Yoshie H., Kudo A., Maeda T. (2004). Immunohistochemical localization of periostin in tooth and its surrounding tissues in mouse mandibles during development. Anat Rec Pt A Discov Mol Cell Evol Biol.

[63] Takeshita S., Kikuna R., Tezuka M., Amann E. (1993). Osteoblast-specific factor-2: cloning of a putative bone adhesion protein with homology to the insect protein fasciclin I. Biochem J.

[64] Kim J.E., Kim S.J., Lee B.H., Park R.W., Kim K.S., Kim I.S. (2000). Identification of motifs for cell adhesion within the repeated domains of transforming growth factor--induced gene, ig-h3. J Biol Chem.

[65] Clout N., Hohenester E. (2003). A model of FAS1 domain 4 of the corneal protein βig-h3 gives a clearer view on corneal dystrophies. Mol Vis.

[66] Clout N.J., Tisi D., Hohenester E. (2003). Novel fold revealed by the structure of a FAS1 domain pair from the insect cell adhesion molecule Fasciclin I. Structure.

[67] Dubbs J.M., Tabita F.R. (2004). Regulators of nonsulfur purple phototrophic bacteria and the interactive control of CO2 assimilation, nitrogen fixation, hydrogen metabolism and energy generation. FEMS Microbiol Rev.

[68] Roh J.H., Smith W.E., Kaplan S. (2004). Effects of oxygen and light intensity on transcriptome expression in *Rhodobacter sphaeroides* 2.4.1. J Biol Chem.

[69] Arai H., Roh J.H., Kaplan S. (2008). Transcriptome dynamics during the transition from anaerobic photosynthesis to aerobic respiration in *Rhodobacter sphaeroides* 2.4.1. J Bacteriol.

[70] Zeilstra-Ryalls J.H., Kaplan S. (2004). Oxygen intervention in the regulation of gene expression: the photosynthetic bacterial paradigm. Cell Mol Life Sci.

[71] Nakayama J., Cao Y., Horii T., Sakuda S., Akkermans A.D.L., de Vos W.M., Nagasawa H. (2001). Gelatinase biosynthesis-activating pheromone: a peptide lactone that mediates a quorum sensing in *Enterococcus faecalis*. Mol Microbiol.

[72] Puskas A., Greenberg E.P., Kaplan S., Schaefer A.L. (1997). A quorum-sensing system in the free-living photosynthetic bacterium *Rhodobacter sphaeroides*. J Bacteriol.

[73] Novak R.T., Gritzer R.F., Leadbetter E.R., Godchaux W. (2004). Phototrophic utilization of taurine by the purple nonsulfur bacteria *Rhodopseudomonas palustris* and *Rhodobacter sphaeroides*. Microbiol.

[74] McCleary W.R., Stock J.B. (1994). Acetyl phosphate and the activation of two-component response regulators. J Biol Chem.

[75] Kappler U., Huston W.M., McEwan A.G. (2002). Control of dimethylsulfoxide reductase expression in *Rhodobacter capsulatus*: the role of carbon metabolites and the response regulators DorR and RegA. Microbiol.

[76] Wolfe A.J. (2005). The acetate switch. Microbiol Mol Biol Rev.

[77] Comolli J.C., Carl A.J., Hall C., Donohue T. (2002). Transcriptional activation of the *Rhodobacter sphaeroides* cytochrome *c2* gene P2 promoter by the response regulator PrrA. J Bacteriol.

[78] Ferré A., de la Mora J., Ballado T., Camarena L., Dreyfus G. (2004). Biochemical study of multiple CheY response regulators of the chemotactic pathway of *Rhodobacter sphaeroides*. J Bacteriol.

[79] Kirndӧrfer M., Jäger A., Klug G. (1998). Integration host factor affects the oxygen-regulated expression of photosynthesis genes in *Rhodobacter capsulatus*. Mol Gen Genet.

[80] Hemschemeier S.K., Kirndӧrfer M., Hebermehl M., Klug G. (2000). DNA binding of wild type RegA protein and its differential effect on the expression of pigment binding proteins in *Rhodobacter capsulatus*. J Mol Microbiol Biotechnol.

[81] Schindel H.S., Bauer C.E. (2016). The RegA regulon exhibits variability in response to altered growth conditions and differs markedly between *Rhodobacter* species. Microb Genom.

[82] Romagnoli S., Packer H.L., Armitage J.P. (2002). Tactic responses to oxygen in the phototrophic bacterium *Rhodobacter sphaeroides* WS8N. J Bacteriol.

[83] Hallenbeck P.C., Abo-hashesh M., Ghosh D. (2012). Strategies for improving biological hydrogen production. Bioresour Technol.

[84] Lee I.-H., Park J.Y., Kho D.H., Kim M.-S., Lee J.K. (2002). Reductive effect of H_2_ uptake and poly-β-hydroxybutyrate formation on nitrogenase-mediated H_2_ accumulation of *Rhodobacter sphaeroides* according to light intensity. Appl Microbiol Biotechnol.

[85] Kars G., Gündüz U. (2010). Towards a super H_2_ producer: improvements in photofermentative biohydrogen production by genetic manipulation. Int J Hydrogen Energy.

[86] Liu T., Li X., Zhou Z. (2010). Improvement of hydrogen yield by *hupR* gene knock-out and *nifA* gene overexpression in *Rhodobacter sphaeroides* 6016. Int J Hydrogen Energy.

[87] Ryu M.H., Hull N.C., Gomelsky M. (2014). Metabolic engineering of *Rhodobacter sphaeroides* for improved hydrogen production. Int J Hydrogen Energy.

[88] Rey E.F., Heiniger K.E., Harwood S.C. (2007). Redirection of metabolism for biological hydrogen production. Appl Environ Microbiol.

[89] Laguri C., Phillips-Jones M.K., Williamson M.P. (2003). Solution structure and DNA binding of the effector domain from the global regulator PrrA (RegA) from *Rhodobacter sphaeroides*: insights into DNA binding specificity. Nucleic Acids Res.

[90] Mao L., Mackenzie C., Roh J.H., Eraso J.M., Kaplan S., Resat H. (2005). Combining microarray and genomic data to predict DNA binding motifs. Microbiol.

